# Novel method for immunofluorescence staining of mammalian eggs using non-contact alternating-current electric-field mixing of microdroplets

**DOI:** 10.1038/srep15371

**Published:** 2015-10-19

**Authors:** Shirasawa Hiromitsu, Kumagai Jin, Sato Emiko, Kabashima Katsuya, Kumazawa Yukiyo, Sato Wataru, Miura Hiroshi, Nakamura Ryuta, Nanjo Hiroshi, Minamiya Yoshihiro, Akagami Yoichi, Terada Yukihiro

**Affiliations:** 1 Department of Obstetrics and Gynecology, Akita University Graduate School of Medicine, Akita, Japan; 2 Akita Industrial Technology Center, Akita, Japan; 3 Division of Clinical Pathology, Akita University Graduate School of Medicine, Akita, Japan; 4 Department of Thoracic Surgery, Akita University Graduate School of Medicine, Akita, Japan

## Abstract

Recently, a new technique was developed for non-catalytically mixing microdroplets. In this method, an alternating-current (AC) electric field is used to promote the antigen–antibody reaction within the microdroplet. Previously, this technique has only been applied to histological examinations of flat structures, such as surgical specimens. In this study, we applied this technique for the first time to immunofluorescence staining of three-dimensional structures, specifically, mammalian eggs. We diluted an antibody against microtubules from 1:1,000 to 1:16,000, and compared the chromatic degree and extent of fading across dilutions. In addition, we varied the frequency of AC electric-field mixing from 5 Hz to 46 Hz and evaluated the effect on microtubule staining. Microtubules were more strongly stained after AC electric-field mixing for only 5 minutes, even when the concentration of primary antibody was 10 times lower than in conventional methods. AC electric-field mixing also alleviated microtubule fading. At all frequencies tested, AC electric-field mixing resulted in stronger microtubule staining than in controls. There was no clear difference in a microtubule staining between frequencies. These results suggest that the novel method could reduce antibody consumption and shorten immunofluorescence staining time.

Immunofluorescence staining is a very important procedure for evaluating the functions of cells, including human and murine eggs^1^,^2^,^3^. In particular, this method allows evaluation of molecular binding and enzymatic reactions within eggs. In common practice, the antibodies used in immunofluorescence are diluted as specified in a supplier-provided data sheet, and the antibody reaction usually proceeds for 1–2 hours at room temperature or overnight in order to optimize antibody concentration and incubation time^4^,^5^. The staining method, in which eggs are placed in a droplet of a few tens of microliters containing diluted antibody, has not changed for a long time. Among the antibodies typically used for these purposes, many are precious or expensive. Assuming that a clear immunofluorescence staining image can be obtained, it is desirable to limit consumption of the antibody and shorten the overall time required for incubation. As a solution to this problem, a recently developed method for promoting the antigen–antibody reaction takes advantage of the non-contact mixing effect of a microdroplet subjected to an alternating-current (AC) electric field^6^,^7^,^8^,^9^. Microdroplet mixing has also been attempted using acoustic actuation, electrowetting, and magnetic actuation, but an effective method for mixing microdroplets was not established until recently^10^,^11^,^12^. This technique, using AC electric-field mixing, makes it possible to transform the shape of a microdroplet by placing it between polar plates and automatically switching a constant voltage on and off at uniform intervals (Fig. 1A). By switching the voltage in this manner, the shape of the microdroplet between the electrodes is made irregular. Consequently, flow occurs inside of the droplet, and this transformation of the droplet’s shape allows mixing to occur (Fig. 1B, Supplementary Movie 1). Prototype devices that use this technique have been constructed, and a few reports describing their use have been published^7^,^8^,^9^. These reports show that immunostaining of thinly sliced perioperative merotomy specimens from lung cancer can be shortened from 120 minutes to 19 minutes. Recently, this method was adopted for intraoperative pathological diagnosis of brain tumors^9^; the device used for this purpose, the Histo-tek® R-IHC® (Sakura Finetek Japan, Tokyo, Japan), has been commercially available since May 2014. The Histo-tek® is shown in Fig. 2A, and the object glass placed on the electrods is shown in Fig. 2B. Previously, this device has only been applied to histological analysis of flat structures like immunostained slices of surgery specimens. In earlier reports of AC electric-field mixing, a droplet of approximately 150 μl was used for staining of specimens. By contrast, in this study we focused on mixing of microdroplets on the order of 15 μl or smaller, and then applied this technique for the first time to immunofluorescence staining of three-dimentional structures, specifically, mammalian eggs. We placed murine eggs inside microdroplets containing antibody, and hypothesized that transformation of the microdroplet would result in three-dimensional mixing, ultimately resulting in effective immunofluorescence staining.

Previous reports have also suggested that pH level, the presence of oxygen, and time are associated with fading of immunofluorescence staining^13^,^14^. We hypothesized that because the antigen–antibody reaction is promoted by electric-field mixing, this method might relieve fading. Hence, we examined the degree of fading after samples underwent AC electric-field mixing.

In this study, we investigated whether immunofluorescence staining of murine eggs by AC electric-field mixing could have the potential leading to reduce consumption of antibody and shorten staining time. We also assessed whether there was a difference in microtubule immunofluorescence staining in the presence or absence of the AC electric field.

## Results

Following AC electric-field mixing, there were no clear differences in microtubule morphology between the control group and the AC electric-field mixing group (Fig. 3A). Similarly, AC electric-field mixing did not destroy the form of murine blastocytes (Supplementary Fig. 1).

Images obtained with a high-speed camera revealed the changes in the form of the microdroplet at each frequency (Fig. 4). There was a difference in the irregularity of the droplet between frequencies (Supplementary Movies 2,3,4,5,6,7).

We show the results of immunofluorescence staining at each antibody concentration in Fig. 3A. With a staining time of only 5 minutes for both the primary and secondary antibody, it was possible to confirm the presence of microtubules in the control group at a primary antibody dilution of 1:4,000. However, at dilutions of 1:8,000 and 1:16,000, staining became weak, and observation became difficult in the control group. By contrast, in the 5 Hz AC electric-field mixing group, staining was stronger than in the control group, and observation was relatively easy because there was little fading. The difference relative to the control group was particularly clear at 1:8,000 and 1:16,000 (i.e., extremely low antibody concentrations). In Fig. 3B, we show the chromatic degree at each frequency at a dilution of 1:16,000; the degree of fading at 5, 21, 32, and 46 Hz was weak relative to the control group. There were no clear macroscopic differences in chromatic degree between frequencies at this dilution.

We showed the Relative Intensity (RI) for each concentraion of primary antibody and each frequency as a box-and-whisker plot in Fig. 5; the associated data are summarized in Supplementary Table 1. In all cases, the mean RI was higher in the AC electric-field mixing group than in the control group. The RI level was statistically significantly higher at dilutions of 1:2,000, 1:8,000, and 1:16,000 at the time of the first observation in the 5 Hz AC electric-field mixing group. By the second observation, the RI level was significantly higher (1.4–2.7-fold) at all concentrations in the 5 Hz AC electric-field mixing group. At a dilution of 1:16,000, the 5, 21, 32, and 46 Hz AC electric-field mixing groups had significantly higher RI levels relative to the control group, in both the first and second observations. In both the first and second observation, there were no statistically significant differences between frequencies.

## Discussion

**Figure 1:**
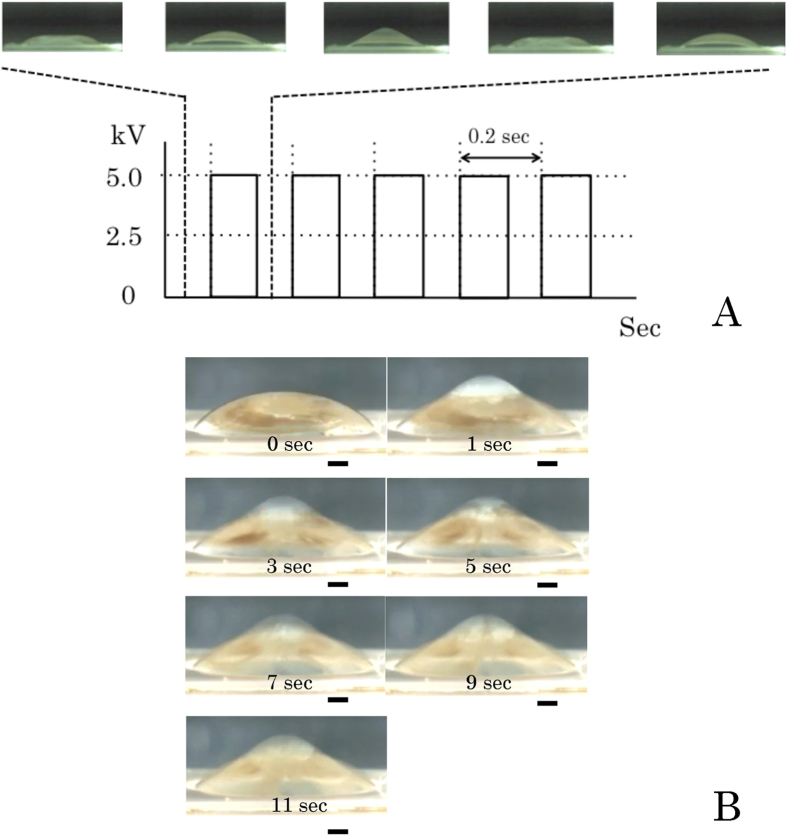
(**A**) Changes in the form of the microdroplet as the voltage was switched on and off in a time series. A frequency of 5 Hz is shown as an example. (**B**) We put 1 μl of FG beads® in 14 μl distilled water and performed AC electric field mixing. Using a high-speed camera, we demonstrated that FG beads® were mixed within the microdroplet. Scale bar: 1 mm.

We showed that AC electric-field mixing increased the intensity of immunofluorescence staining of microtubules. Of course, in such an evaluation, it is necessary to consider individual differences in the eggs. Also, microtubules were stained to some extent in only 5 minutes when eggs were simply left to rest in droplets containing antibody. At a high antibody concentration (e.g., a dilution of 1:1,000) , the antibody reacted sufficiently with antigen even in the control group; therefore, there was no apparent difference between the conditions. RI tended to increase in the mixing group at the first observation stating at a dilution of 1:2,000. When the antibody concentration was low, staining after 5 minutes was weak, and the degree of fading was strong in the control group. However, when antibody was used at a concentration 10-fold lower than usual, microtubule staining could be achieved after 5 minutes of AC field mixing. Moreover, fading was minimized in samples subjected to AC electric-field mixing, facilitating the second observation (conducted 48 hours after the first). Various frequencies resulted in different degrees of transformation of the droplet, as shown in Fig. 1 and Supplementary Movies 2–7. However, no clear difference in chromatic degree was observed between the 5, 21, 32, and 46 Hz groups. This result contradicts previous reports suggesting that different frequencies of AC electric fields result in different degrees of mixing^7^. However, it is possible that differences in frequency influence chromatic strength as a function of the target size. In these experiments, we stained microtubles, which are relatively large, but, when staining a small protein like cohesin which resides near the chromosomes, a differences in frequency may become more important.

Transformation of microdroplets has been attempted previously using vibration, acoustic actuation, microwave irradiation, and physical methods^10^,^11^,^12^,^15^. It is well known that liquids do not mix easily in microfluidics^12^. Therefore, methods for mixing the interiors of microdroplets have been developed as so-called “labs-on-a-chip”. However, no previous report has described immunofluorescence staining of three-dimentional stuructures, such as eggs, in microdroplets. One advantage of electric-field mixing is that the interior of the microdroplet can be mixed non-catalytically, and an important advantage of AC field mixing without physical contact is that is we can mix the droplet nondestructively. To date, the efficiency of immunochemical staining using AC electric field mixing has only been reported in three English-language publications^7^,^8^,^9^. In those studies, thinly sliced pathology specimens were fixed on object glass. By contrast, in this study, we placed murine eggs in microdroplets and mixed them; i.e., the eggs moved within the microdroplets. This study provides the first demonstration of the use of this technique to achieve effective immunofluorescence staining of objects like mammalian eggs. Furthermore, past studies applied AC electric field mixing to pathology specimens for the purpose of effective intraoperative diagnosis. Therefore, one of the main objectives of past studies was to determin whether they could shorten the time required to perform the staining protocol. In our this study, we examined a utility of AC electric field mixing when we performed immunofluorescence staining in a short time on the condition that we diluted an antibody. In addition, we were able to conduct the immunofluorescence staining in murine blastocytes by AC electric field mixing without negatively affecting the sample’s morphology (Supplementary Fig. 1). Threfore, we believe that this method will be applicable in reproductive medicine for staining samples at various stages of development from unfertilized egg to blastocyst.

As shown in Figs 3 and 5, we were able to perform the immunofluorescence staining effectively in a short time by AC electric field mixing. Beacause this is a new technique, the underlying principles have not been examined in detail in past reports^7^,^8^,^9^. Even for methods in which pathology specimens are immunostained using ultrasound and microwaves^16^,^17^, the mechanism underlying the promotion of the antigen-antibody reaction is not completely understood. The antigen-antibody reaction in conventional tissue staining is caused by Brownian movement; therefore, conventional methods of staining require longer intervals of time^18^. Vibration of the droplet is thought to promote the antigen-antibody reaction by increasing the number of opportunities for each antibody molecule to encounter its specific antigen^16^. Exposure to the AC electric causes mixing to occur inside the microdroplet (Fig. 1 and Supplementary Movie 1). Consequently, the number of physical contacts between the egg and antibodies increase and the antigen-antibody reaction is enhanced. Promotion of the antigen-antibody reaction allows staining with highly diluted antibody and may reduce fading.

It is also important to consider the effect of electroporation, in which an aperture in the cell membrane is created by application of an electric current. This technique has been applied to introduction of mRNA^19^ and immunostaining of murine eggs^20^. We put a microdroplet of 15 μl on the object glass, and them suspended eggs in the microdroplet. The microdroplet was separated from the electrode by the object glass and air, which are both insulator, and was therefore not in direct contact with the electrode. Because the droplet was in a dielectric and equipotential environment, and no current was generated in the droplet, no electric current flowed into the eggs. We showed that an electric current did not flow through the inside of the microdroplet during AC electric field mixing (Supplementary Fig. 2 and Supplementary Movie 8). Therefore, we think that electroporation did not occur in this experiment. Instead, the transformation of the microdroplet was caused by attraction of the microdroplet to the electrode side by Coulomb forces when the electric field was applied.

Finally, we considered whether this method of immunofluorescence staining with mixing leads to reduce costs relative to conventional staining methods. To date, several methods for effective immunostaining have been used, including microwave^15^,^16^, ultrasound^17^, and high quality reagents^21^. Past reports focused on the reduction in staining time, and did not devote attention to reduction of cost. In this study, we considerd only chromatic efficiency of microtubule. As shown in Fig. 3, RI of AC electric field mixing group was higher than the RI of the non-mixing group in an antibody concentration of 2-fold in both the first and second observation. Thus, we may reduce antibody quantity by approximately 50% in a short staining time (e.g., 5 minutes for each antibody). Future examination is necessary to determine in detail how much each antibody concentration can be reduced when using AC electric field mixing. When we think about the effect in other antigens, there may be a difference in a utility of AC electric field mixing by the presence site of the desired antigen. In this study, we performed AC electric field mixing for each antibody for only five minutes, but it is necessary to determine whether extending the mixing time enables more effective staining. Although additional studies are required, we predict that AC electric field mixing may reduce the consumption of the antibody to some extent.

In summary, we applied AC electric-field mixing for the first time to murine eggs. Because the antigen–antibody reaction was promoted by mixing, staining was intense and fading was minimal; consequently, observation was possible even at very low concentrations of antibody. These findings indicated that this method may has the potential to reduce the quantiy of antibody used and shorten the staining time. We predict that this technique could be applied in multiple ways to studies of eggs and other cell types.

## Online Methods

### Egg collection and fixation

All procedures for murine care and use were carried out in accordance with the guidelines and approved by the Animal Research Committee of the Akita University, Japan (Permit number: 26-1-40).

Murine eggs were obtained from imprinting control region (ICR) mice at 4–7 weeks of age. Mice were stimulated for egg collection according to a previous report^5^,^22^. Briefly, we injected 7.5 IU of pregnant mare serum gonadotropin (PMSG) (ASKA Pharma, Tokyo, Japan) into the murine abdominal cavity, sacrificed the mouse 48 hours later, and then collected germinal vesicle (GV) eggs. We fixed eggs after culturing them for at least 7 hours in culture medium containing MG-132 to obtain meiosis I stage eggs. The fixation method was described previously^13^. Briefly, we incubated the eggs in 2% paraformaldehyde + KB buffer (20 mM Tris-HCl, pH 7.5, and 150 mM NaCl) for 30 minutes. After washing, the eggs were incubated for 15 minutes in 0.2% Triton X-100 + KB-BSA (20 mM Tris-HCl, pH 7.5, 150 mM NaCl, 0.1% BSA). After washing, eggs were incubated overnight at 4 °C in KB-BSA.

### Egg staining

Eggs were subjected to immunofluorescence staining to detect microtubules, as previously described^23^. The primary antibodies were mouse monoclonal anti–acetylated tubulin antibody (1.0 mg/ml) (Sigma–Aldrich, St. Louis, MO, USA) and mouse monoclonal anti–β-tubulin antibody (2.0 mg/ml) (Sigma–Aldrich), and the secondary antibody was Alexa Fluor® 488–conjugated goat anti–mouse IgG (2.0 mg/ml) (Life Technologies, Carlsbad, CA, USA). Supplier data sheets for all three antibodies recommend that they should be used for immunofluorescence staining at a dilution of 1:200–1:1,000, and previous studies often used them at these concentrations^5^,^24^. For these experiments, primary antibodies were diluted 1:1,000, 1:2,000, 1:4,000, 1:8,000, or 1:16,000, and the dilution of the secondary antibody was fixed at 1:1,000. Eggs were mounted in VECTASHIELD® Mounting Medium with DAPI (Vector Laboratories, Burlingame, CA, USA) to allow observation of chromosomes and prevent fading. For each dilution, we stained six eggs.

### AC electric-field mixing and control group

**Figure 2:**
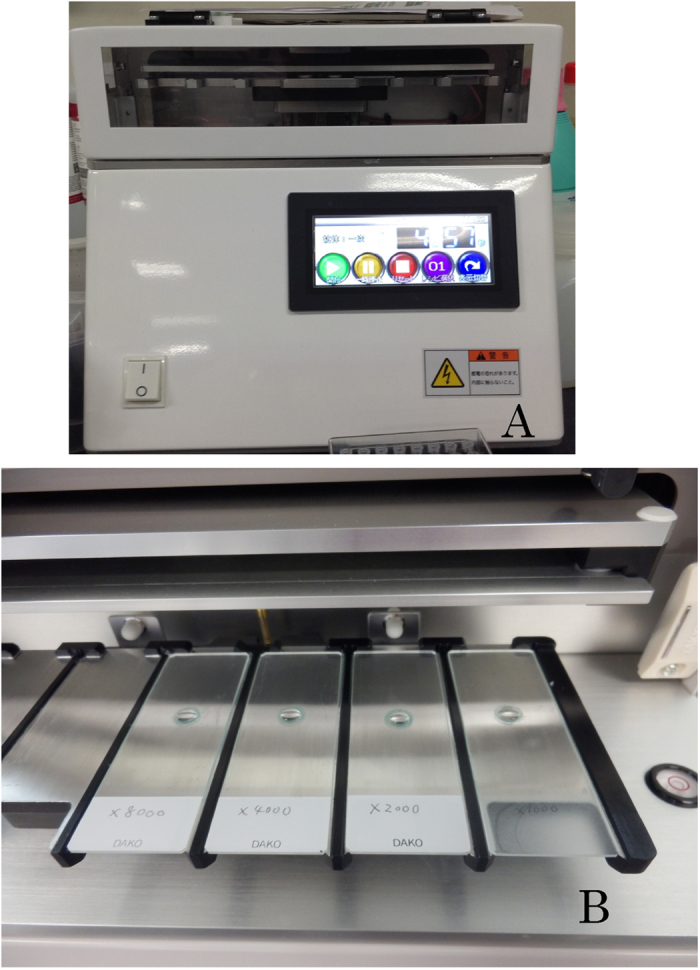
(**A**) The Histo-Tek® R-IHC®. (**B**) A 15-μl droplet containing antibody on the microscope slide between the electrodes.

**Figure 3:**
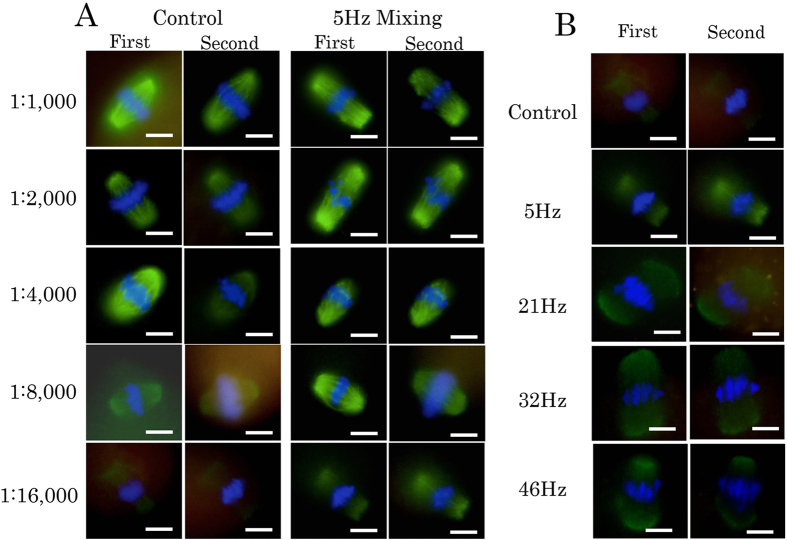
(**A**) Comparison between control and AC electric-field mixing at 5 Hz, for each antibody concentration. Immunofluorescence staining of eggs is shown for the first and second observations.(**B**) Immunofluorescence staining of eggs is shown for the 1:16,000 dilution at each frequency. The second observation was carried out 48 hours after the first observation. Green, microtubules; blue, DAPI. Scale bar: 10 μm.

**Figure 4:**
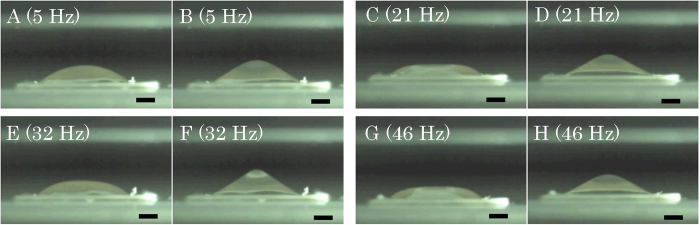
Changes in the irregularity of the microdroplet at 5 Hz (**A,B**), 21 Hz (**C,D**), 32 Hz (**E,F**), and 46 Hz (**G,H**) using a high-speed camera. Scale bar: 1 mm.

**Figure 5:**
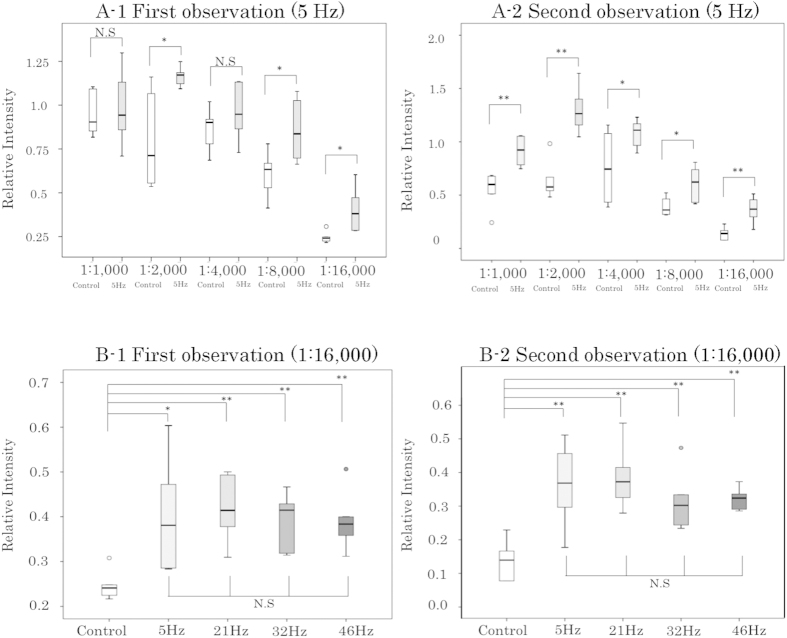
Box-and-whisker plots of Relative Intensity for each condition. The bottom and top of each box mark the 25th and 75th percentiles, respectively. The whiskers are drawn down to the 5th percentile and up to the 95th. Points below and above the whiskers represent outliers. *P < 0.05, **P < 0.01, two-sample t-test or Welch’s t-test. N.S.: not significant. (**A1**) First observation comparing 5 Hz AC electric-field mixing with the control. (**A2**) Second observation comparing 5 Hz AC electric-field mixing with the control. (**B1**) First observation comparing AC electric-field mixing at 5 Hz, 21 Hz, 32 Hz, and 46 Hz with a non-mixed control at an antibody concentration of 1:16,000. (**B2**) Second observation comparing AC electric-field mixing at 5 Hz, 21 Hz, 32 Hz, and 46 Hz with a non-mixed control at an antibody concentration of 1:16,000. Comparisons between the four AC electric-field mixing groups were subjected to the Bonferroni correction for multiple comparisons.

AC electric-field mixing was performed using a Histo-Tek® R-IHC® (Sakura Finetek Japan, Tokyo, Japan), shown in Fig. 2. The theory and technique of electric-field mixing have been described in detail in previously published papers^6^,^7^,^8^,^9^. Briefly, we first placed the microscope slide between the electrodes and applied a uniform voltage of 5 kV between them, generating an electric field between the electrodes, as shown in Fig. 1A,B. The voltage was turned on and off at regular intervals, resulting in transformation of the droplet’s shape, as shown in Fig. 1A for the case of 5 Hz. At this time, we applied a voltage of 5.0 kV to the droplet at frequency of 5–46 Hz. We visualized the state of mixing in the microdroplet using nanobeads (Fig. 1B). We put 1 μl of FG beads® (Tamagawa Seiki, Tokyo, Japan) in 14 μl of distilled water and performed AC electric field mixing. Figure 4 shows the shapes of the microdroplet observed at each frequency (Supplementary Movies 2–7). We drew a circle 5 mm in diameter with a Dako Pen® (Dako, Carpinteria, CA, USA) to prevent the collapse of the droplet on the microscope slide, created a droplet (15 μl) containing primary or secondary antibody, placed six eggs inside the droplet, and then performed non-contact AC electric-field mixing.

After a 5-min period of electric field mixing in the presence of primary antibody at room temperature, we washed the eggs in KB buffer for 1 minute, moved each egg to a droplet containing secondary antibody, and performed electric field mixing for an additional 5 minutes. The AC electric field mixing took place at 5 Hz for dilution of 1:8,000 to 1:1,000. In 1:16,000 the least dense, 21 Hz, 32 Hz, 46 Hz underwent AC field mixing in addition to 5 Hz order to investigate the influence of different frequencies. In the control group, eggs were left to rest in primary antibody for 5 minutes without AC field mixing. It was assumed that the secondary antibody only left an egg at rest similarly in the control group for 5 minutes.

### Observation and comparison

Eggs were observed and analyzed on a DP73 microscope (Olympus, Tokyo, Japan) equipped with a digital color and monochrome camera. The objective lens was a 100 × oil-immersion lens. The imaging software was cellSens® Standard (Olympus). We limited observation of each egg to <1 minute to prevent fluorescence fading. Focus matches the whole and we only adjusted gain so that microtubule contrast became clear; otherwise, we did not perform any type of image editing. For the first observation, eggs were observed immediately after mounting in VECTASHIELD® Mounting Medium with DAPI. The second observation, to determine the degree of fading, was carried out 48 hours after first observation. During the first and second observations, we kept eggs in the dark at 4 °C. The immunofluorescence staining protocol is summarized in Table 1. The two groups were compared to determine whether there was a difference in microtubule morphology, microtubule staining intensity, or degree of fading.

### Image analysis

To evaluate the intensity of microtubules staining quantitatively, we used the ImageJ software (version 1.48 for Mac OS X)^24^,^25^. Chromosomes were stained using VECTASHIELD® Mounting Medium with DAPI. The intensity of chromosome staining was not influenced by the presence or absence of electric-field mixing, and the dilution of primary antibody never affected the intensity of chromosome staining. Fluorescence intensities of microtubules and chromosome were measured manually for each outline^22^. Relative intensity (RI) of microtubules was determined as (mean area density of green signal in microtubules/ mean area density of blue staining in chromosome), according to previous reports^24^,^26^. We compared the RI between the AC electric-field mixing group and the control group.

### Statistical analysis

Data were analyzed by two-sample t-test or Welch’s t-test with Levene’s test using the SPSS software version 21.0 (SPSS Inc., Chicago, IL, USA). A value of probability (P) < 0.05 or <0.01 was considered statistically significant. We applied Bonferroni correction for multiple comparisons between groups.

## Additional Information

**How to cite this article**: Hiromitsu, S. *et al.* Novel method for immunofluorescence staining of mammalian eggs using non-contact alternating-current electric-field mixing of microdroplets. *Sci. Rep.* **5**, 15371; doi: 10.1038/srep15371 (2015).

## Supplementary Material

Supplementary Movie 1

Supplementary Movie 2

Supplementary Movie 3

Supplementary Movie 4

Supplementary Movie 5

Supplementary Movie 6

Supplementary Movie 7

Supplementary Movie 8

Supplementary Information

## Figures and Tables

**Table 1:** Immunofluorescence staining protocol.

	Control group	Mixing group at each frequency
Fixation of eggs	Room temperature	Room temperature
30 min	30 min	
Primary antibody	Rest in the droplet	AC electric field mixing
5 min	5 min	
Washing	Room temperature	Room temperature
1 min	1 min	
Secondary antibody	Rest in the droplet	AC electric field mixing
5 min	5 min	
Washing	Room temperature	Room temperature
1 min	1 min	
Mounting of eggs	Room temperature	Room temperature
VECTASHIELD®	VECTASHIELD®	
30 min	30 min	
First observation	Room temperature	Room temperature
<1 min per egg	<1 min per egg	
Store of specimens	Dark room at 4 °C	Dark room at 4 °C
48 h	48 h	
Second observation	Room temperature	Room temperature
for fading	<1 min per egg	<1 min per egg

AC, alternating-current; VECTASHIELD®, VECTASHIELD® Mounting Medium with DAPI.
