# Corrigendum: Novel method for immunofluorescence staining of mammalian eggs using non-contact alternating-current electric-field mixing of microdroplets

**DOI:** 10.1038/srep31941

**Published:** 2016-09-07

**Authors:** Hiromitsu Shirasawa, Jin Kumagai, Emiko Sato, Katsuya Kabashima, Yukiyo Kumazawa, Wataru Sato, Hiroshi Miura, Ryuta Nakamura, Hiroshi Nanjo, Yoshihiro Minamiya, Yoichi Akagami, Yukihiro Terada

Scientific Reports 5: Article number: 1537110.1038/srep15371; published online: 10 19 2015; updated: 09 07 2016

The original version of this Article contained errors in the spelling of the authors Hiromitsu Shirasawa, Jin Kumagai, Emiko Sato, Katsuya Kabashima, Yukiyo Kumazawa, Wataru Sato, Hiroshi Miura, Ryuta Nakamura, Hiroshi Nanjo, Yoshihiro Minamiya, Yoichi Akagami and Yukihiro Terada, which were incorrectly given as Shirasawa Hiromitsu, Kumagai Jin, Sato Emiko, Kabashima Katsuya, Kumazawa Yukiyo, Sato Wataru, Miura Hiroshi, Nakamura Ryuta, Nanjo Hiroshi, Minamiya Yoshihiro, Akagami Yoichi and Terada Yukihiro

In addition, the ‘How to cite this article’ section quoted an incorrect abbreviation for Hiromitsu Shirasawa. These errors have now been corrected in the PDF and HTML versions of the Article.

