# Supplementary material for: Novel method for immunofluorescence staining of mammalian eggs using non-contact alternating-current electric-field mixing of microdroplets
Source: Sci Rep. 2015 Oct 19;5:15371. doi: 10.1038/srep15371 (PMC4609987; doi:10.1038/srep15371)
Supplement: Supplementary Information [file srep15371-s9.pdf]

Novel method for immunofluorescence staining of mammalian eggs using non-contact alternating-current electric-field mixing of microdroplets

Authors
Shirasawa Hiromitsu, Kumagai Jin, Sato Emiko, Kabashima Katsuya, Kumazawa Yukiyo, Sato Wataru, Miura Hiroshi, Nakamura Ryuta, Nanjo Hiroshi, Minamiya Yoshihiro, Akagami Yoichi, Terada Yukihiro

Supplementary Figures S1 and S2
Supplementary Table 1

Supplementary Figure S1

Immunofluorescence staining of Connexin43 in mouse blastocysts by AC electric field mixing

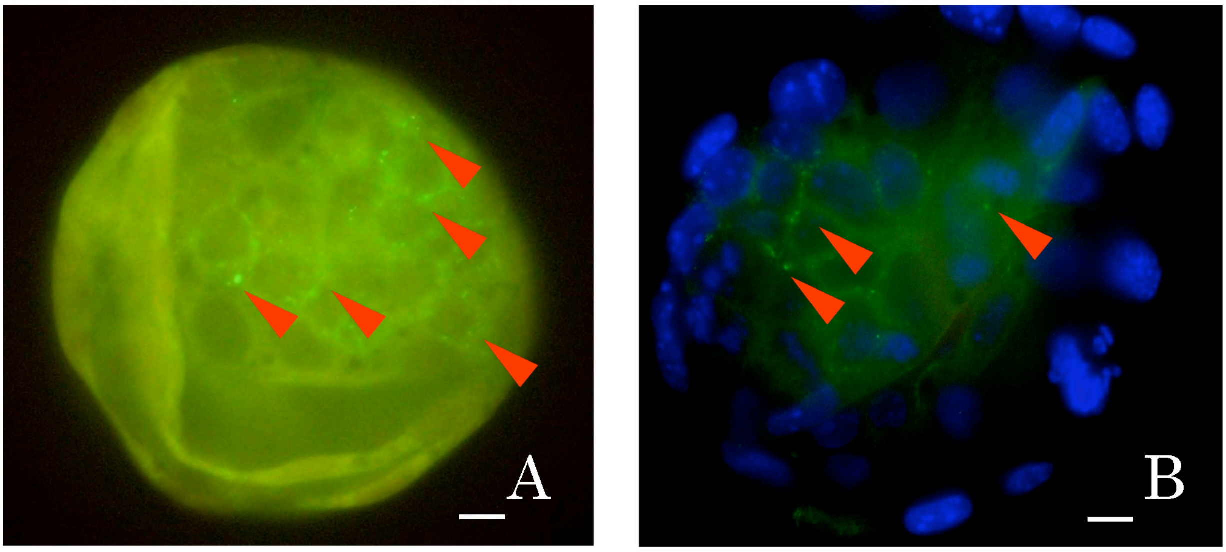

AC electric field mixing for immunofluorescence staining was applied to fixed murine oocytes. Anti-Connexin43 (20369933, ZYMED, Sun Francisco, CA, USA) diluted 1:5,000 was used as the primary antibody. Alexa Fluor 488–conjugated goat mouse IgG (Invitrogen, Carlsbad, CA, USA) diluted 1:1,000 was used as the secondary antibody. To allow observation of chromosomes, the cells were stained dilute Hoechst 33342. AC electric field mixing at 5 Hz was performed according to the protocol shown in Table 1. The mixing time was for 5 minutes for each antibody. Staining results of two representative blastocysts as shown. (A) Only Connexin43 was stained. Green, Connexin43 (red arrowhead). (B) Connexin43 and chromosomes were stained. Green, Connexin43 (red arrowhead); Blue, chromosomes. Scale bar: 10 µm.

Supplementary Figure S2

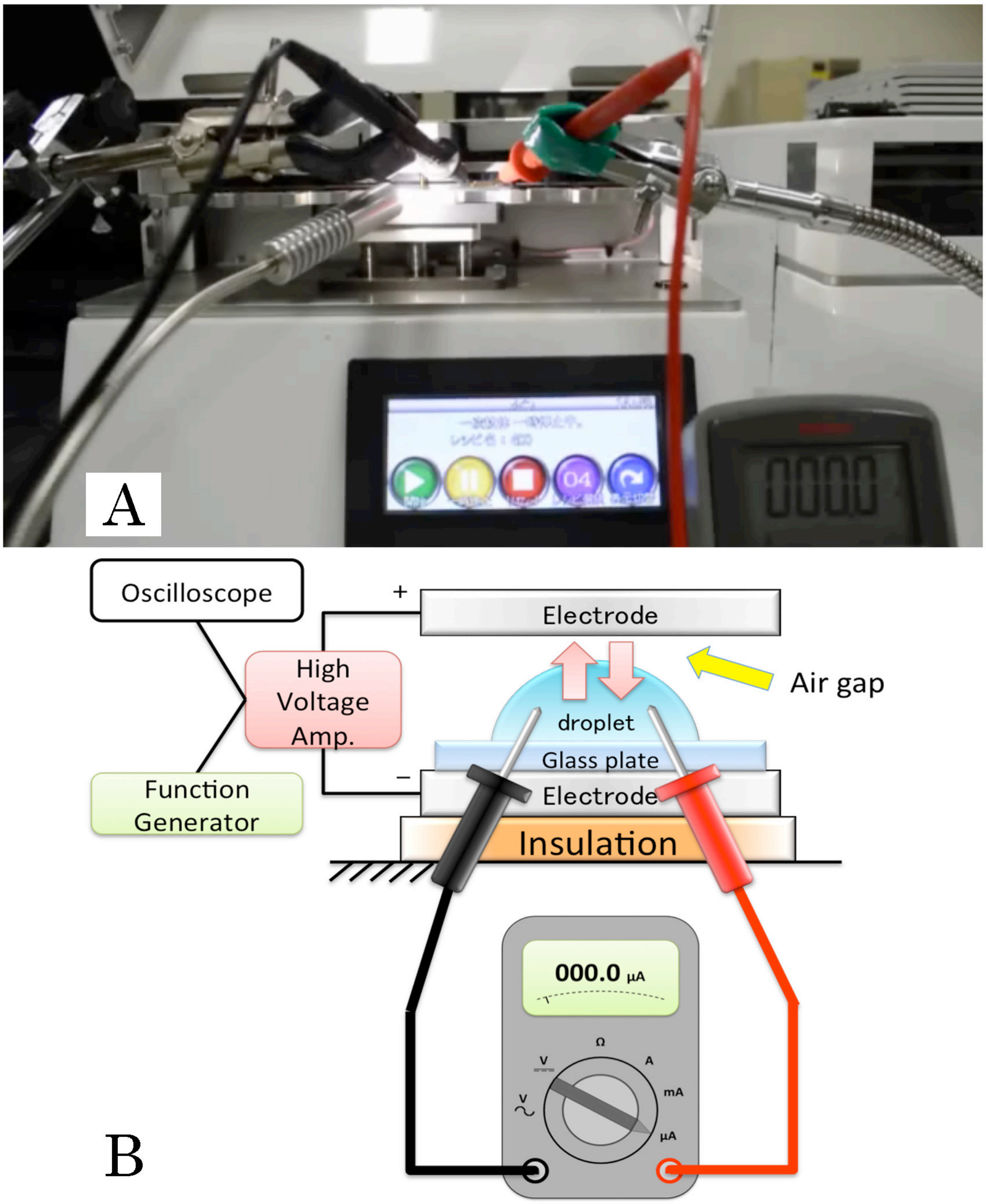

Measurement of current in the microdroplet during AC electric field mixing

While conducting AC electric field mixing, we investigated whether an electric current flowed through the microdroplet. (A) Apparatus for measuring electric current. The tester probes (black and red) were inserted into a microdroplet of phosphate-buffered saline. (B) Schematic view of the amperometry method; see also Supplementary Movie 8. During the test, the numerical value of the ammeter did not change; therefore, no electric current flowed through the microdroplet. This illustration was drawn by N.R.

Supplementary Table 1

Relative intensity of each condition.

Dilution	Observation		RI mean	S.D	P value
1:1,000	First	Control	0.954	0.136	0.783
		5Hz	0.988	0.231	
	Second	Control	0.529	0.207	0.006**
		5Hz	0.914	0.130	
1:2,000	First	Control	0.790	0.263	0.017*
		5Hz	1.161	0.053	
	Second	Control	0.650	0.198	< 0.001**
		5Hz	1.296	0.204	
1:4,000	First	Control	0.868	0.118	0.279
		5Hz	0.959	0.157	
	Second	Control	0.758	0.326	0.048*
		5Hz	1.080	0.126	
1:8,000	First	Control	0.609	0.127	0.019*
		5Hz	0.856	0.175	
	Second	Control	0.396	0.092	0.030*
		5Hz	0.607	0.167	
1:16,000	First	Control	0.248	0.036	0.036*
		5Hz	0.401	0.133	
		21Hz	0.418	0.072	0.001**
		32Hz	0.393	0.062	0.001**
		46Hz	0.390	0.065	0.002**
	Second	Control	0.138	0.064	0.009**
		5Hz	0.362	0.132	
		21Hz	0.385	0.092	0.001**
		32Hz	0.315	0.087	0.004**
		46Hz	0.322	0.032	< 0.001**

RI, relative intensity; S.D, standard deviation; P value, probability value

*P<0.05, **P<0.01, 2-sample t-test or Welch's t-test.
